# A case of BNT162b2 COVID‐19 vaccine‐associated fulminant myocarditis in a very elderly woman

**DOI:** 10.1002/ccr3.6161

**Published:** 2022-09-05

**Authors:** Kaishi Otsuka, Takashi Matsuo, Takashi Ishimatsu, Atsuki Fukae, Takuro Hamamoto, Koji Oku, Masahiro Ito

**Affiliations:** ^1^ National Hospital Organization Nagasaki Medical Center Nagasaki Japan

**Keywords:** cardiogenic shock, COVID‐19, heart failure, myocarditis

## Abstract

Coronavirus disease 2019 (COVID‐19) vaccination is reportedly safe and effective. The histologic features of post‐COVID‐19 vaccination myocarditis are unknown. We present a case of a 77‐year‐old Japanese woman diagnosed with eosinophilic myocarditis using endomyocardial biopsy, 7 days after the second dose of BNT162b2 COVID‐19 vaccine. Steroid pulse therapy was effective.

## Abstract 2

Myocarditis following COVID‐19 vaccination is a rare complication, and most cases are mild. However, the present case was a severe one in an elderly woman who responded remarkably to steroid pulse therapy. Eosinophilic myocarditis should be suspected in patients with heart failure after COVID‐19 vaccination.

## 1 INTRODUCTION

There have been several reports of myocarditis after BNT162b2‐mRNA or mRNA‐1273 coronavirus disease 2019 (COVID‐19) vaccination.^1^, ^2^ However, few reports have focused on the pathological diagnosis of myocarditis after BNT162b2 vaccination. Here, we present a case of eosinophilic myocarditis after BNT162b2 vaccination that was successfully treated using steroid pulse therapy.

## 2 CASE HISTORY

A 77‐year‐old Japanese woman presented to our hospital with generalized fatigue, 7 days after receiving the second dose of the BNT162b2 COVID‐19 vaccine. Her medical history was notable for dyslipidemia without heart disease, and there was no family history of cardiovascular disease.

## 3 DIFFERENTIAL DIAGNOSIS, INVESTIGATIONS, AND TREATMENT

On admission, her blood pressure was 100/67 mmHg; heart rate, 75 beats per minute, and oxygen saturation on room air, 98%. Her heart and lung sounds were normal, and she had no abdominal tenderness or lower extremity edema. Laboratory test results indicated renal dysfunction (blood urea nitrogen level: 31.2 [normal value: 8–20] mg/dl and creatinine level: 1.30 [0.46–0.79] mg/dl) and liver dysfunction (total bilirubin level: 0.7 [0.4–1.5] mg/dl, aspartate aminotransferase level: 97 [13–30] U/L, and alanine aminotransferase level: 59 [7–23] U/L).

Her brain natriuretic peptide (BNP), troponin T, creatine kinase (CK), CK‐MB, and C‐reactive protein levels were elevated to 1661 pg/ml, 8.4 ng/ml, 532 U/L, 71 U/L, and 6.6 mg/L, respectively. The results of her COVID‐19 polymerase chain reaction testing were negative. Further, investigation results for parvovirus B19, mycoplasma, Epstein–Barr virus, adenovirus, influenza virus, and herpes simplex viruses 1 and 2 were also negative.

**FIGURE 1:**
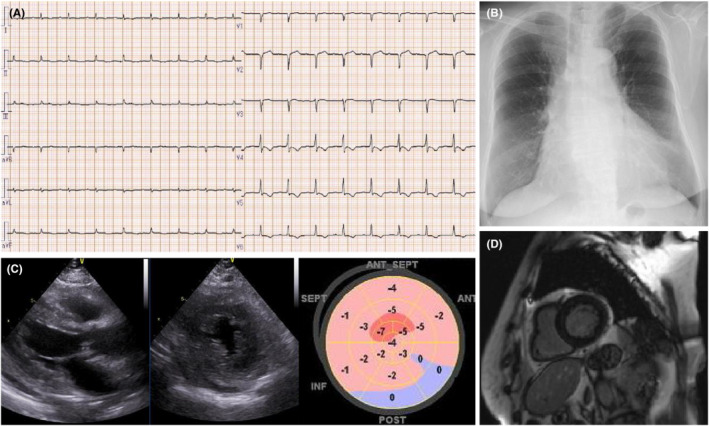
Results of investigations at admission. (A) Electrocardiogram shows a heart rate of 102 beats per minute, first‐degree atrioventricular block, low voltage across all the limb leads, and inverted T waves in leads V3–V6. (B) Chest radiograph shows cardiomegaly with a cardiothoracic ratio of 55%. (C) Echocardiogram shows left ventricular hypertrophy and diffuse left ventricular hypokinesis. LVDd: 37 mm, LVDs: 29 mm, IVST: 13 mm, LVPWD: 13 mm, LVEF: 41%, and GLS: −2 to −5%. (D) Contrast‐enhanced cardiac magnetic resonance image obtained on Day 19 of admission shows no delayed gadolinium enhancement because it was performed after steroid pulse therapy. ANT, anterior wall; GLS, global longitudinal strain; INF, inferior wall; IVST, interventricular septum thickness; LVDd, left ventricular internal dimension in diastole; LVDs, left ventricular end‐diastolic dimension; LVEF, left ventricular ejection fraction; LVPWD, left ventricular posterior wall diameter; POST, posterior wall; SEPT, septal wall

Electrocardiography performed on admission showed a first‐degree atrioventricular block, low voltage in all the limb leads, and inverted T waves in leads V3–V6 (Figure 1A). Her chest radiograph showed cardiomegaly with a cardiothoracic ratio of 55% (Figure 1B). Transthoracic echocardiography revealed left ventricular hypertrophy (interventricular septum thickness: 13 mm and thickness of the posterior wall of the left ventricle: 13 mm), diffuse left ventricular hypokinesis with a left ventricular ejection fraction (LVEF) of 41% (Simpson's method), and a decreased global longitudinal strain of −2% (Figure 1C).

Our differential diagnoses included acute coronary syndrome, heart failure, and cardiomyopathy.

**FIGURE 2:**
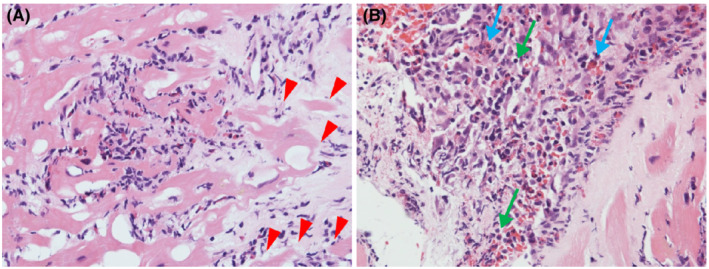
Pathological results of the first endomyocardial biopsy. Histopathological specimen shows inflammatory cells largely composed of eosinophils and mononuclear inflammatory cells; myocyte necrosis is also seen. (A) hematoxylin and eosin stain. (B) hematoxylin and eosin stain. Red arrows: These show the vanishing of cardiomyocytes. Blue arrows: These show the infiltration of eosinophils. Green arrows: These show the infiltration of lymphocytes

After admission, her blood pressure gradually decreased to 90/60 mmHg on Day 2. Additionally, her urine output decreased to 150 ml per 8 h, despite adequate infusion. Therefore, we performed an emergency cardiac catheterization. Swan–Ganz catheterization indicated a low output syndrome and mild pulmonary hypertension (Table S1). We subsequently performed intra‐aortic balloon pump (IABP) insertion and started continuous intravenous infusion of dopamine (3 μg/kg/min) and dobutamine (6 μg/kg/min). In lieu of normal coronary angiographic findings, we performed a right ventricular endomyocardial biopsy for rapid pathological diagnosis using a frozen section. Histologically, inflammatory infiltrates were observed at the interstitial spaces and at the interface between the cardiomyocytes and loose fibrous stroma. Inflammatory cells largely composed of eosinophils and mononuclear cells and myocyte necrosis were observed (Figure 2). We made a diagnosis of post‐COVID‐19 vaccination eosinophilic myocarditis and started methylprednisolone pulse therapy (1000 mg/day).

Her condition improved dramatically in response to the therapy. On Day 2 of treatment, her LVEF improved from 41% to 65%. Cardiac output also improved from 2.5 to 4.0 L/min, and cardiac index improved from 1.6 to 2.8 L/min/m^2^ (Table S1). Her urine output increased from 150 to 800 ml per 8 h without diuretics, and subsequently, her renal and liver functions normalized. The IABP was removed on Day 5 of admission, and the patient was weaned off inotropes by Day 9.

After 3 days of methylprednisolone pulse therapy, the patient was switched to oral prednisolone 60 mg per day, which was gradually tapered.

Contrast‐enhanced cardiac magnetic resonance imaging (MRI) on Day 19 of admission showed no delayed gadolinium enhancement or abnormal T1 and T2 mapping (Figure 1D).

On Day 26, we performed a second right ventricular endomyocardial biopsy and histologically confirmed that acute inflammation had subsided and tissue remodeling was ongoing. Some lymphocytic infiltration, hemosiderin deposition, and fibrosis were observed in the injured interstitium. Eosinophilic infiltration was not observed.

**FIGURE 3:**
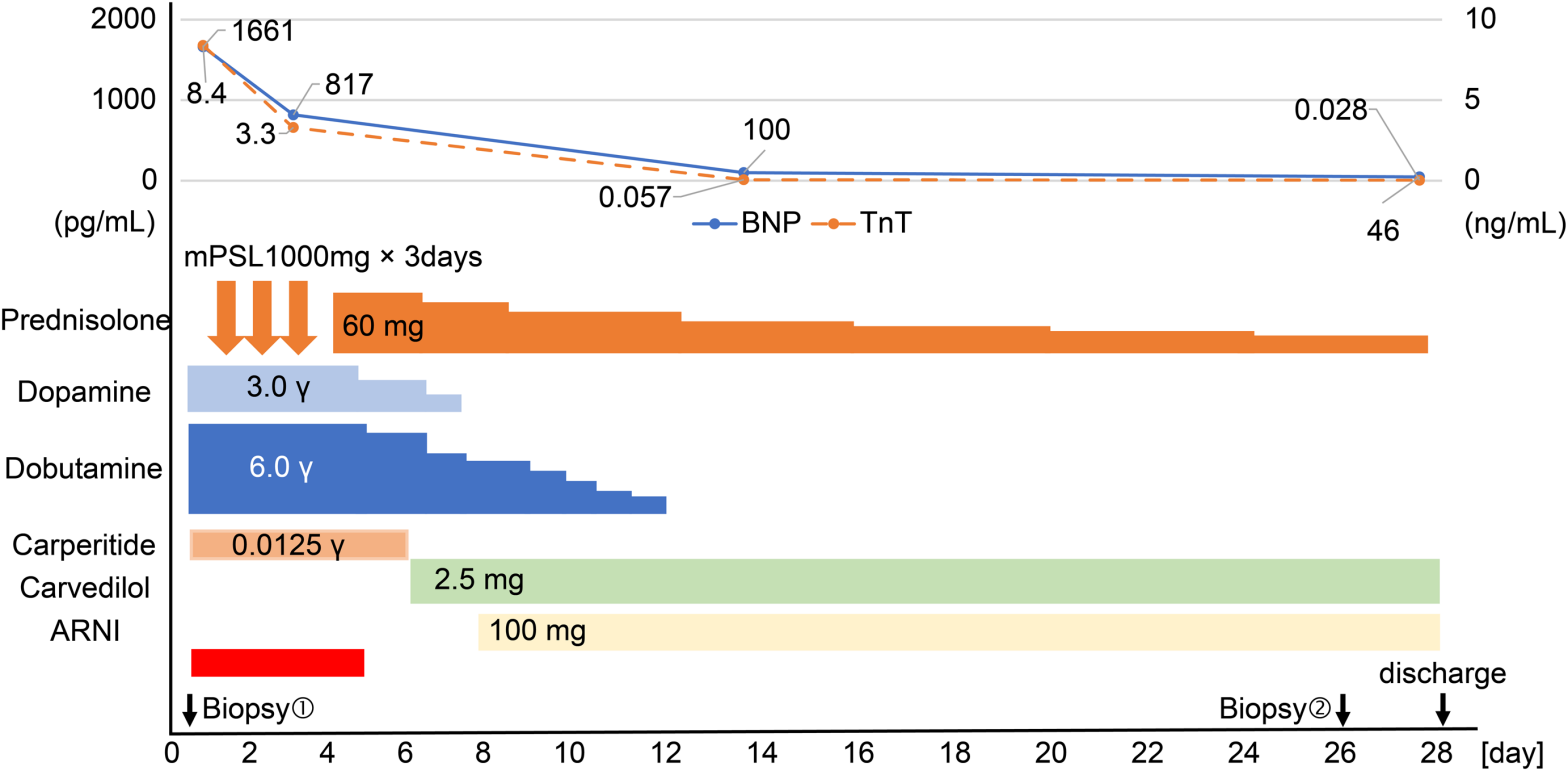
Patient's clinical course. BNP, brain natriuretic peptide; mPSL, methylprednisolone; TnT, troponin T; γ, μg/kg/min

On Day 28, the patient was discharged following improvement in the LVEF (66%), BNP (46 pg/ml), and troponin T (0.028 ng/ml) levels. At discharge, we prescribed carvedilol (2.5 mg per day), sacubitril/valsartan (100 mg per day), and prednisolone (15 mg per day) (Figure 3).

## 4 OUTCOME AND FOLLOW‐UP

Two months after discharge, the patient was asymptomatic with normal cardiac function. Prednisolone was tapered and discontinued at 3 months post‐discharge. Carvedilol (2.5 mg per day) and sacubitril/valsartan (ARNI, 100 mg per day) were continued.

## 5 DISCUSSION

Myocarditis is most commonly caused by viral infections, although idiopathic, autoimmune, and hypersensitivity‐related myocarditis have also been reported.^1^ Most patients with myocarditis have cold‐like symptoms (e.g., chills, fever, headache, muscle aches, and fatigue) and develop signs of heart failure (e.g., arrhythmia, hypotension, and pulmonary congestion). In this case, the patient presented with generalized fatigue, and she developed exacerbation of heart failure after hospital admission.

There have been several reports of myocarditis after mRNA COVID‐19 vaccination. Lee et al. reported that 14 US military personnel developed myocarditis after receiving mRNA COVID‐19 vaccines, which included both the mRNA‐1273 (Moderna, Cambridge, MA, USA) and BNT162b2‐mRNA (Pfizer‐BioNTech; New York, NY, USA and Mainz, Germany) formulations.^2^ Staff et al. reported that out of over 5 million people who received the mRNA COVID‐19 vaccine, 62 developed myocarditis, of whom two people (a 22‐year‐old woman and a 35‐year‐old man) died.^3^ Most individuals who developed myocarditis were males aged <30 years. Generally, the prevalence of myocarditis after mRNA COVID vaccination has been reported as approximately 0.00001%. However, according to Staff et al., it may be as high as 0.00005% among men aged 16–30 years.^3^ As with our patient, most patients in those studies experienced myocarditis after receiving the second dose of the vaccine.

Cases of post‐vaccination myocarditis or pericarditis have been reported in the literature, particularly after smallpox vaccination,^4^ for which the incidence was reported to be 200 times higher than that in the general population.^5^ There have been reports of myopericarditis after vaccination against human papillomavirus, influenza, tetanus, and hepatitis B,^6^ although its prevalence is lower than that of myocarditis and pericarditis.^4^

The pathophysiology of post‐vaccination myocarditis and pericarditis remains unclear. Furthermore, there has been only one reported case of eosinophilic myocarditis after tetanus toxoid immunization diagnosed by endomyocardial biopsy, and the report suggested an underlying mechanism of nonadaptive immune‐mediated injury or hypersensitivity reaction.^7^

Almost all previously reported cases of myocarditis after mRNA COVID‐19 vaccination were diagnosed using cardiac MRI, and only one patient underwent endomyocardial biopsy, although there was no pathological evidence of myocarditis.^8^ To the best of our knowledge, this is the first case in which endomyocardial biopsy was used to diagnose acute eosinophilic myocarditis after the patient received the second dose of mRNA COVID‐19 vaccine.

The optimal treatment for myocarditis after COVID‐19 vaccination is yet to be determined. According to previous reports, most patients with myocarditis after mRNA COVID‐19 vaccination have good outcomes.^2^, ^3^ Although steroid therapy, including methylprednisolone pulse therapy,^9^, ^10^ has been used in previous cases, the rationale for its use was unclear because endomyocardial biopsy had not been performed. Our patient was diagnosed with eosinophilic myocarditis, which is an indication for steroid therapy. In fact, steroid pulse therapy can dramatically improve cardiac function in such patients. Endomyocardial biopsy is important for determining the etiology of myocarditis, even in post‐vaccination cases because the result may affect treatment selection. Moreover, our report suggests that vaccine‐related myocarditis may occur even in elderly women, although it has been previously reported that it mostly occurs in male patients under 50 years of age.^2^, ^3^, ^8^, ^9^, ^10^

Very few patients with myocarditis after COVID‐19 vaccination underwent endomyocardial biopsy and steroid therapy. In this case, we were able to diagnose eosinophilic myocarditis using endomyocardial biopsy. However, it is difficult to prove an association between myocarditis and COVID‐19 vaccination, and this could be a limitation of our report. In addition to myocarditis, other adverse events of COVID‐19 vaccination have been reported, including optic neuropathy, Guillain–Barre syndrome, and thrombocytopenia.

Further studies are needed to determine the exact mechanisms and optimum treatments for patients with myocarditis after COVID‐19 vaccination.

## 6 CONCLUSION

Eosinophilic myocarditis should be suspected in patients with heart failure after COVID‐19 vaccination, and endomyocardial biopsy is recommended for these patients.

## AUTHOR CONTRIBUTIONS

Kaishi Otsuka involved in writing the paper. Takashi Matsuo involved in drafting the article. Takuro Hamamoto involved in analysis and interpretation of data. Atsuki Fukae involved in revising the manuscript critically for important intellectual content. Takashi Isimatsu involved in acquisition of data. Koji Oku involved in conception and design of study. Masahiro Ito involved in providing pathological images and analyzing pathological findings.

## FUNDING INFORMATION

This report received no specific grant from any funding agency in the public, commercial, or not‐for‐profit sectors.

## CONFLICT OF INTEREST

The authors declare that they have no conflict of interest.

## ETHICAL APPROVAL

We did not obtain IRB approval because this is a case report.

## CONSENT

Written informed consent was obtained from the patient to publish this report in accordance with the journal's patient consent policy.

## Supporting information


Table S1

## Data Availability

Data sharing not applicable to this article as no datasets were generated or analysed during the current study.
