# Supplementary material for: A case of BNT162b2 COVID‐19 vaccine‐associated fulminant myocarditis in a very elderly woman
Source: Clin Case Rep. 2022 Sep 5;10(9):e6161. doi: 10.1002/ccr3.6161 (PMC9445258; doi:10.1002/ccr3.6161)
Supplement: Supplementary file 1 — Table S1 [file CCR3-10-0-s001.docx]

**Supporting Table 1. Swan–Ganz catheter findings on days 2, 3, and 26 of admission**

|  | **Day 2** | **Day 3** | **Day 26** |
| --- | --- | --- | --- |
| CO/CI (Fick) | 2.5/1.7 | 4.0/2.8 | 3.0/2.1 |
| CVP (mmHg) | 13 | 7 | 8 |
| mPAP (mmHg) | 21 | 22 | 13 |
| mPAWP (mmHg) | 17 |  | 5 |

CI: cardiac index, CO: cardiac output, CVP: central venous pressure, mPAP: mean pulmonary artery pressure, mPAWP: mean pulmonary artery wedge pressure
